# Supplementary material for: Chromothripsis during telomere crisis is independent of NHEJ, and consistent with a replicative origin
Source: Genome Res. 2019 May;29(5):737–49. doi: 10.1101/gr.240705.118 (PMC6499312; doi:10.1101/gr.240705.118)
Supplement: Supplemental Material [file supp_gr.240705.118_Supplemental_file_1.zip › contigs/annotated_contigs/DB114/contig.3.DB114_length_693_mean_cov_5.19480519481.docx]

**DB114_length_693_mean_cov_5.19480519481**

```
ATTTATGAATAGGGACCAGAGAGCCCCTTCCATGTGACGCCTATGACAGCACCACATCAGCTTCAAGGAGAGGTGTCAGCAGAACGTAA
 >chr12:20293673-20293980 - E=7e-164
CTCATATTTCAAACCTCTCCCAGCTCTATAATCTCACAATCATTGTTCCTCTTTTTCTTCAAGGCCTCACCTTGAAACCAACCCAGTCC

AATTGTCCCATGGGTTCCTATCTTCAGAGAAAATTCTTAATCTCTCTATGAGGACTTAGAAGGTTATTTTCAATAATCTCTTTTGTCTT

TTACTTGCCAAATTTCTTATATTTCTTCTGTTTTTTTCT|T|CATGATAACACTTTGGATTTATATCTCAAAAAGTAACACGTTTTGAA
                                        >chr12:20291590-20291938 + E=1e-194
CTACTTTAACACTTCTATAAACTAAAAAACTTAGTAGTTTACAAAACCTTATTGCCTTATTTAACAGATACCCAAACTGAGTTAAAGAT

AAATTAAGTGACTTCCGCATGGTCAAACTGTGGTAGATTTAGCATTATTTGTTTTTCCTAACTCTGTTTCAACACCTTAGTATCCATTA

CCTGCCTTCTGAAAATAAAAGGATATGGCTACGAACTAAACACACTGAAGGTGAATGATATATTTTCTCCCCAGAAACTATTTGTATGA

TGTAGGGATTAGTAACAGGAAAACTAAAAT|TTA|AAGTAGTTCAAAACGTGTTACTTTTTGAGATATAAATCC                               >chr12:20291605-20291647 - E=2e-10
```
